# Quantitative capillary refill time predicts sepsis in patients with suspected infection in the emergency department: an observational study

**DOI:** 10.1186/s40560-019-0382-4

**Published:** 2019-05-06

**Authors:** Oi Yasufumi, Naoto Morimura, Aya Shirasawa, Hiroshi Honzawa, Yutaro Oyama, Shoko Niida, Takeru Abe, Shouhei Imaki, Ichiro Takeuchi

**Affiliations:** 1 Emergency and Critical Care Medical Center, Yokohama Municipal Citizen’s Hospital, 56 Okazawacho, Hodogayaku, Yokohama City, Kanagawa 240-8555 Japan; 2 Department of Emergency Medicine, Yokohama City University School of Medicine, Yokohama, Japan; 3 Department of Acute Medicine, Graduate School of Medicine, The University of Tokyo, Tokyo, Japan; 4 Advanced Critical Care and Emergency Center, Yokohama City University Medical Center, Yokohama, Japan

**Keywords:** Sepsis, Organ dysfunction scores, Systemic inflammatory response syndrome, Emergency service, Triage

## Abstract

**Background:**

Outcomes in emergent patients with suspected infection depend on how quickly clinicians evaluate the patients and start treatment. This study was performed to compare the predictive ability of the quantitative capillary refill time (Q-CRT) as a new rapid index versus the quick sequential organ failure assessment (qSOFA) score and the systemic inflammatory response syndrome (SIRS) score for sepsis screening in the emergency department.

**Methods:**

This was a multicenter, observational, retrospective study of adult patients with suspected infection. The area under the curve (AUC) of receiver operating characteristic curve analyses and multivariate analyses were used to explore associations of the Q-CRT with the qSOFA score, SIRS score, and lactate concentration.

**Results:**

Of the 75 enrolled patients, 48 had sepsis. The AUC, sensitivity, and specificity of Q-CRT were 0.74, 58%, and 81%, respectively; those for the qSOFA score were 0.83, 66%, and 100%, respectively; those for the SIRS score were 0.61, 81%, and 40%, respectively, for SIRS score; and those for the lactate concentration were 0.76, 72%, and 81%, respectively. We found no statistically significant differences in the AUC between the scores. We then combined the Q-CRT and qSOFA score (Q-CRT/qSOFA combination) for sepsis screening. The AUC, sensitivity, and specificity of Q-CRT/qSOFA combination were 0.82, 83%, and 81%, respectively.

**Conclusions:**

In this study, Q-CRT/qSOFA combination had better sensitivity than the qSOFA score alone and better specificity than the SIRS score alone. There was no significant difference in accuracy between Q-CRT/qSOFA combination and the qSOFA score or lactate concentration. The ability of the Q-CRT to predict sepsis may be similar to that of the qSOFA score or serum lactate concentration; therefore, measurement of the Q-CRT may be an alternative for invasive measurement of the blood lactate concentration in evaluating patients with suspected sepsis.

**Electronic supplementary material:**

The online version of this article (10.1186/s40560-019-0382-4) contains supplementary material, which is available to authorized users.

## Background

Death from sepsis occurs in one patient every several seconds worldwide. Sepsis is a serious condition that affects people of any age. In the initial examination of patients with infection, it is important to determine whether sepsis is present because appropriate whole-body management must be started early after onset [1, 2]. Since the first definition of sepsis as a systemic inflammatory response syndrome (SIRS) by Bone et al. [3] in 1992, this concept has been incorporated into diagnostic criteria [4]. In 2015, a new definition of sepsis (Sepsis-3) and diagnostic criteria were published [5]. The new diagnostic process proposed by Sepsis-3 is used in two situations: diagnosis in patients in the intensive care unit (ICU) and diagnosis in patients outside the ICU (prehospital care, emergency department, and general ward).

Patients in the ICU or other intensive care settings are likely to have a diagnosed or suspected infection, and the rate of sepsis diagnosis increases sharply in patients with a total sequential organ failure assessment (SOFA) score of ≥ 2. In patients outside the ICU, including those managed in the emergency department, sepsis is suspected when assessment according to the new screening criterion, the quick SOFA (qSOFA), is positive for at least two components. If sepsis is suspected, patients are assessed for organ dysfunction. A definitive diagnosis of sepsis is made when the total SOFA score sharply increases to ≥ 2, as can occur in patients in the ICU. Using large-scale data, Seymour et al. [6] examined the relationship between in-hospital mortality and various scoring systems in patients with suspected infection outside the ICU. The qSOFA score had the highest ability to predict in-hospital death. Thus, qSOFA was recommended as a new screening system for sepsis in patients outside the ICU. However, qSOFA consists of a simple combination of indices that emphasize outcomes in patients with sepsis [7], and the component indices are not specific to infection. Therefore, although qSOFA is a good screening tool for identifying patients with sepsis with a poor prognosis, many patients with sepsis may not be identified by qSOFA screening. Other indices that address the drawbacks of qSOFA are needed.

Among various complementary indices, the capillary refill time (CRT) is a parameter of shock. CRT tends to be prolonged in patients with sepsis [8–10]; however, the CRT is determined subjectively, and its assessment lacks objectivity and reproducibility. If quantified and assessed with a high degree of accuracy, the CRT provides a complementary evaluation of circulation, taking full advantage of the characteristic features of CRT measurement.

Using the quantitative CRT (Q-CRT) as a rapid and noninvasive index in combination with a related indicator, we revealed that the arterial blood lactate level was correlated with the Q-CRT using a CRT quantification device in patients in the ICU [11] and that the venous blood lactate level was correlated with the Q-CRT in patients in the emergency department [12]. However, the usefulness of the Q-CRT to predict sepsis among patients with suspected infection is unknown. If the Q-CRT is a reliable index for sepsis, clinicians could make more rapid treatment decisions for patients in the emergency room. Thus, we performed the current study to examine the relationship between the Q-CRT and sepsis compared with the qSOFA and SIRS scores in patients in the emergency department.

## Methods

### Setting

This was a retrospective, multicenter, observational study performed at Yokohama City University Hospital (Yokohama, Japan) and Yokohama Municipal Citizen’s Hospital (Yokohama, Japan). Yokohama City University Hospital’s catchment area is the southern area of Yokohama City, and Yokohama Municipal Citizen’s Hospital’s catchment area is the central area of Yokohama City, which had an estimated population of 3.7 million in 2017.

### Design

This retrospective observational study was performed to examine the relationship between the Q-CRT and sepsis in patients in the emergency department. No sample size calculation was performed because this was an explanatory study. The study was approved by the hospitals’ institutional review boards (Yokohama City University Hospital approval number: B150801105 and Yokohama Municipal Citizen’s Hospital approval number: 17-07-01). All patients or their families provided informed consent to participate in this study.

### Patients

Only patients with a measured Q-CRT and suspected infection in the emergency department were evaluated. The single inclusion criterion was the ability to measure the Q-CRT by a designated physician. The primary exclusion criterion was the inability to measure the Q-CRT because of physical conditions, such as finger injuries causing difficulty attaching the Q-CRT device to the patient. We also excluded patients with physical trauma and those undergoing dialysis. We used a single Q-CRT measuring device, and only two emergency physicians used the device.

### Measurement of Q-CRT

**Fig. 1:**
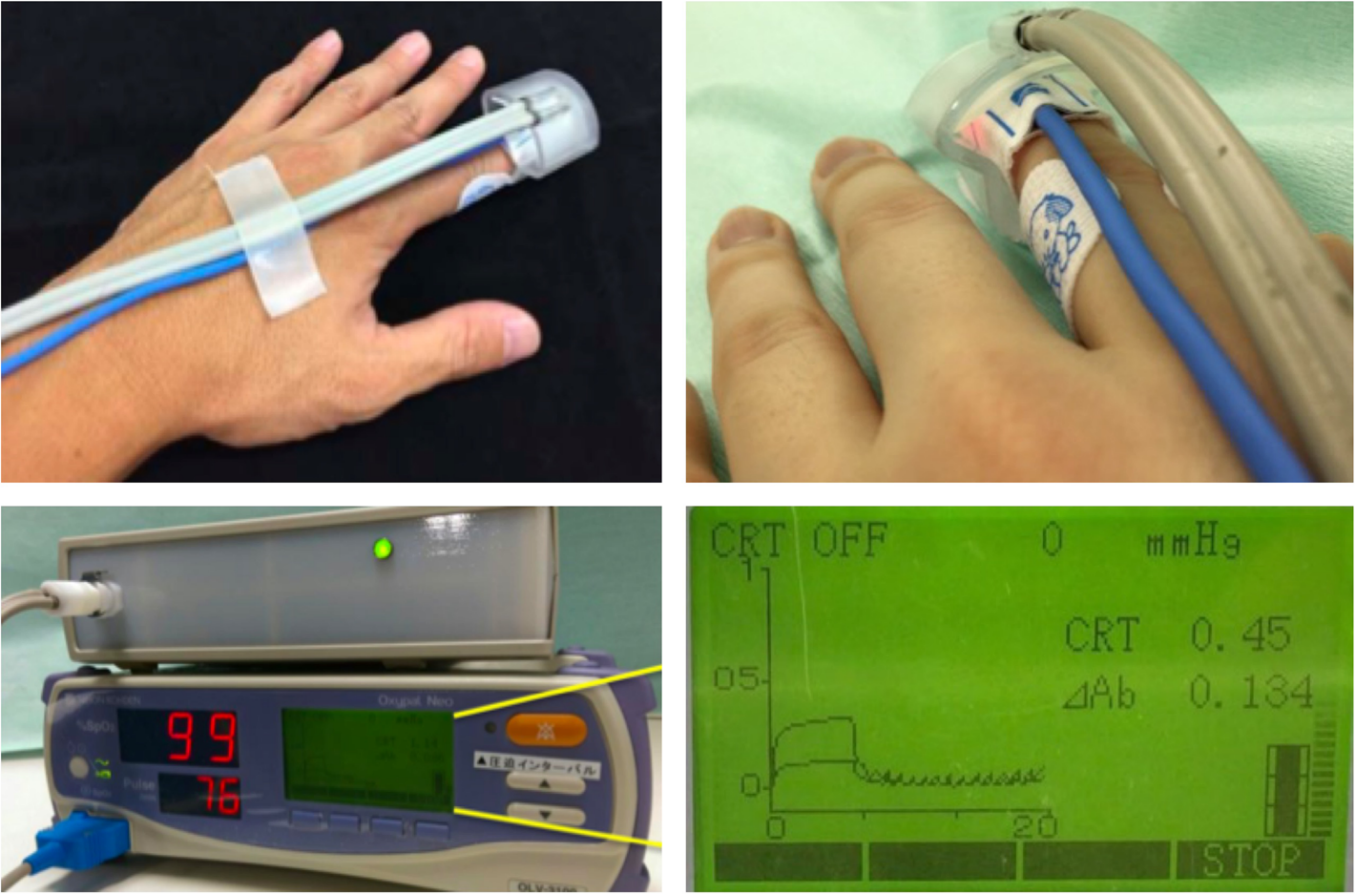
Application of device used to measure quantitative capillary refill time. The device is placed on the finger with a pulse oximeter

**Fig. 2:**
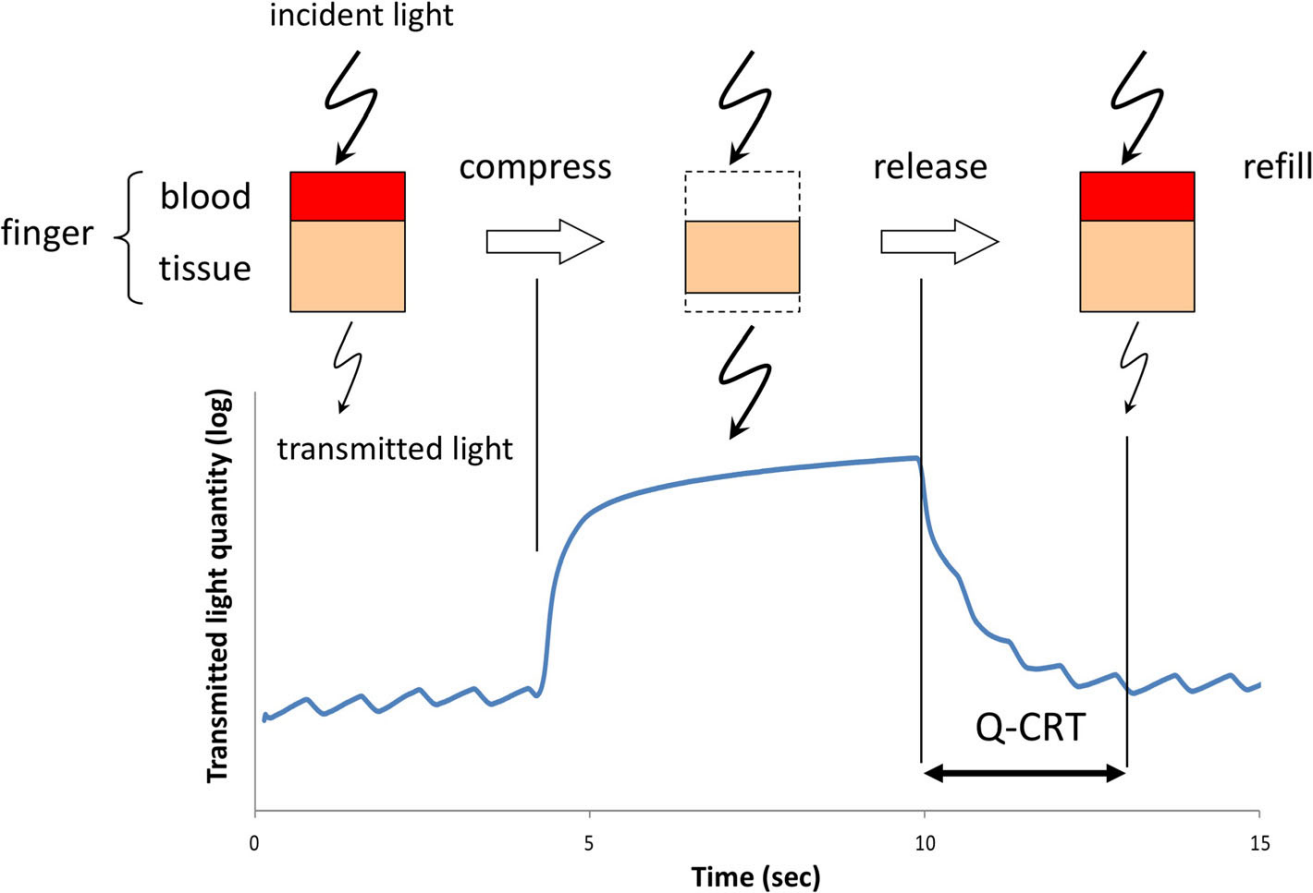
Schematic of quantitative capillary refill time measurements. The quantity of transmitted light obtained by a pulse oximeter equipped with a sensor for hemoglobin saturation of oxygen is related to the blood volume, based on the Lambert–Beer law. Q-CRT, quantitative capillary refill time


Figure 1 and Additional file 1 show the use of the device we used to measure the Q-CRT, and Fig. 2 depicts the schema for Q-CRT measurement. Transmitted light measured by a pulse oximeter equipped with an SpO_2_ sensor is related to the blood volume, based on the Lambert–Beer law [13]. Mechanical pressure of 500 mmHg is applied to the index finger for 5 s. This stops the blood flow, and the transmitted light increases. When the pressure is removed, blood flow restarts, and the transmitted light decreases. In our previous study, we defined the Q-CRT as the time in seconds from the release of pressure to the time point at which the blood flow reaches 90% of the original flow, which was measured for 5 s at the beginning of the test before applying pressure.

Additional file 1 How to use Q-CRT device. The apparatus is placed on the finger with a pulse oximeter (MP4 2190 kb)


### Definition of suspected infection

We used the original paper describing qSOFA to define the initial time of suspected infection [6] as either (1) any culture order followed by administration of an intravenous antibiotic within 72 h or (2) administration of an intravenous antimicrobial followed by a culture order within 24 h. The time of the culture order or intravenous antimicrobial administration was denoted as the time of suspicion of infection, whichever came first.

### Definitions of qSOFA, SIRS, and sepsis

The qSOFA score ranges from 0 to 3, with 1 point awarded for each of the following: systolic blood pressure of ≤ 100 mmHg, respiratory rate of ≥ 22 breaths/min, and change in mental status from baseline. A qSOFA score of ≥ 2 predicts a greater risk of a prolonged ICU stay or increased mortality. The SIRS criteria incorporate the clinical criteria of the Surviving Sepsis Campaign for SIRS [14] and include at least two of the following: heart rate of > 90 beats/min, respiratory rate of > 20 breaths/min, white cell count of < 4000 or > 12,000 cells/mm^3^, and temperature of < 36.0 °C or ≥ 38.3 °C. The definition of sepsis used in this study was based on the clinical criterion of Sepsis-3 [5], namely the presence of life-threatening organ dysfunction caused by a dysregulated host response to infection. Organ dysfunction is represented by a ≥ 2-point increase in the SOFA score. In non-ICU settings, adults with suspected infection are identified as being more likely to have poor outcomes typical of sepsis when they meet at least two of the clinical criteria that constitute the qSOFA score. This study evaluated the predictive performance of the Q-CRT as a screening tool for sepsis in patients with suspected infection in the emergency department.

### Data analysis and statistical methods

Stata (R) 13.1 (StataCorp, College Station, TX, USA) was used for the statistical analyses. Data are presented as median with interquartile range for continuous variables and as number and percentage for categorical variables. Student’s *t* test, the Mann–Whitney *U* test, and the *χ*^2^ test were used for the univariate analysis. Sensitivity, specificity, and the area under the curve (AUC) were calculated for the Q-CRT, qSOFA score of ≥ 2, SIRS score of ≥ 2, and Q-CRT > cutoff + qSOFA score of ≥ 2 to compare the ability of the scores to predict sepsis. AUCs were compared using the DeLong test, and a *p* value of < 0.05 was considered statistically significant (Additional file 2).

## Results

**Fig. 3:**
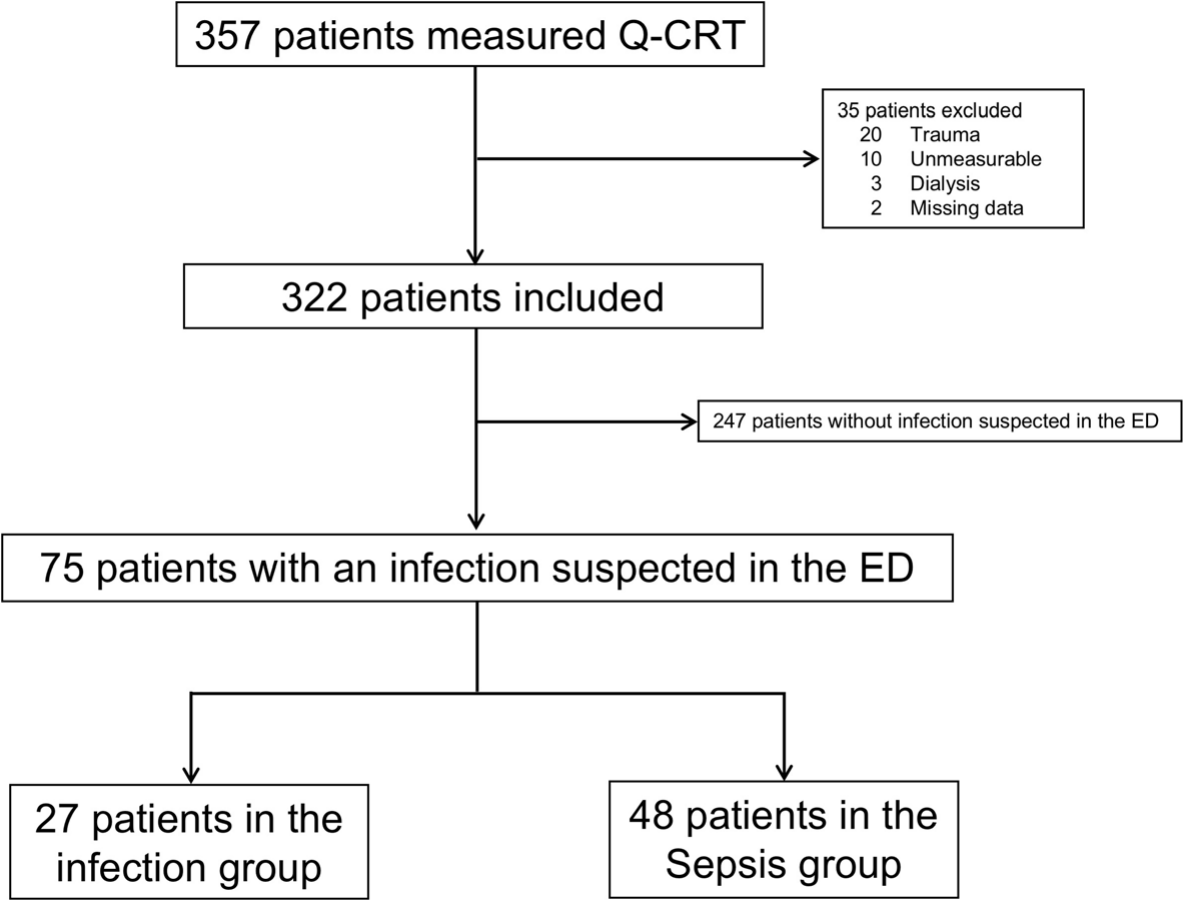
Study flow chart. ED, emergency department; Q-CRT, quantitative capillary refill time. Infection group: infected patients without organ dysfunction. Sepsis group including septic shock: infected patients with organ dysfunction

In the Yokohama City University Hospital, 1323 patients were taken to the emergency department by ambulance from November 2015 to March 2017. We were able to measure the Q-CRT in 286 patients (21.6%), and 31 had suspected infection. In the Yokohama Municipal Citizen’s Hospital, 1152 patients were taken to the emergency department by ambulance from August 2017 to April 2018. We were able to measure the Q-CRT in 71 patients (6.1%), 44 of whom had suspected infection. During the study, we identified 75 patients (21%) with suspected infection, and all were enrolled: 27 (36%) were infected patients without organ dysfunction (infection group) and 48 (64%) were infected patients with organ dysfunction including septic shock (sepsis group) (Fig. 3).

Several differences were noted between the two groups (Table 1). Patients with sepsis were older and had a higher heart rate, creatinine level, lactate level, and Q-CRT. Patients with sepsis also had a low SpO_2_ and platelet count. The sepsis group included a greater proportion of patients with qSOFA and SIRS scores of ≥ 2 and Glasgow coma scale scores of < 15. The respiratory tract was the most common site of infection among patients with sepsis, and the respiratory system was the most frequent index of organ dysfunction in the SOFA scores.

**Table 1:** Baseline characteristics of study participants (*n* = 75)

Characteristics	All patients (*n* = 75)		Infection group (*n* = 27)		Sepsis group (*n* = 48))		*P* value
Sex, no. (%)							
Men^a^	51	(68.0)	19	(70.3)	32	(66.7)	0.741
Women^a^	24	(32.0)	8	(29.6)	16	(33.3)	
Age (years)	77	(63–84)	69	(57–78)	81	(65.5–84.5)	0.016
SBP (mmHg)	130	(115–147)	132	(122–146)	129	(105–150)	0.540
RR (breaths/min)	24	(19–30)	24	(20–26)	25	(18–30)	0.268
HR (beats/min)	105	(91–120)	96	(84–108)	109.5	(96–128)	0.006
GCS< 15, no.(%)^a^	37	(49.3)	3	(11.1)	34	(70.8)	< 0.001
BT (°C)	37.9	(36.8–38.9)	37.8	(36.7–38.4)	37.9	(37.1–39.2)	0.279
SpO_2_ (%)	96	(93–98)	96	(94–98)	95	(91.5–97)	0.034
Site of infection, no. (%)							
Respiratory^a^	35	(46.7)	8	(29.6)	27	(56.2)	0.027
Urinary^a^	14	(18.6)	5	(18.5)	9	(18.7)	0.980
Abdominal^a^	19	(25.3)	10	(37.0)	9	(18.7)	0.080
Cutaneous^a^	5	(6.7)	4	(14.8)	1	(2.0)	0.034
Neurological^a^	1	(1.3)	0	0.0	1	(2.0)	0.450
Bone and joints^a^	1	(1.3)	0	0.0	1	(2.0)	0.450
Others^a^	0	0.0	0	0.0	0	0.0	–
Organ dysfunction (SOFA)							
Respiratory system^a^	40	(53.3)	5	(18.5)	35	(72.9)	< 0.001
Coagulation	18	(24.0)	0	0.0	18	(37.5)	< 0.001
Hepatic system	26	(34.6)	6	(22.2)	20	(41.6)	0.089
Cardiovascular system	10	(13.3)	0	0.0	10	(20.8)	0.011
Cental nervous system	37	(49.3)	3	(11.1)	34	(70.8)	< 0.001
Renal system	22	(29.3)	1	(3.7)	21	(43.7)	< 0.001
Laboratory results							
WBC (/μL)	11,350	(8800–14,300)	12,510	(9620–17,500)	11,030	(8595–13,325)	0.294
Creatinine (mg/dL)	0.92	(0.74–1.28)	0.81	(0.73–0.92)	0.99	(0.77–1.62)	0.006
Bilirubin (mg/dL)	0.9	(0.6–1.4)	0.7	(0.5–1.1)	1.05	(0.6–1.75)	0.067
Platelets (103/μL)	19.4	(13.5–23.4)	21.4	(18.4–25.5)	18.4	(10.8–22.2)	0.009
Lactate (mmol/L)	1.8	(1.24–2.68)	1.4	(1.1–1.6)	1.99	(1.51–3.07)	< 0.001
Q-CRT (s)	3.143	(2.009–5.868)	2.207	(1.591–3.269)	3.923	(2.529–6.694)	< 0.001
Q-CRT > 3.5, no.(%)^a^	33	(44.0)	5	(18.5)	28	(58.3)	0.001
SIRS ≧ 2, no.(%)	55	(73.3)	16	(59.2)	39	(81.2)	0.039
qSOFA ≧ 2, no. (%)	32	(42.6)	0	0.0	32	(66.7)	< 0.001

*Infection group* infected patients without organ dysfunction. *Sepsis group (including septic shock)* infected patients with organ dysfunction. *SBP* systolic blood pressure, *RR* respiratory rate, *HR* heart rate, *GCS* Glasgow Coma Scale score, *BT* body temperature, *SpO2* hemoglobin saturation, *WBC* white blood cell count, *Q-CRT* quantitative capillary refill time, *SIRS* systemic inflammatory response syndrome, *qSOFA* quick sequential organ failure assessment. ^a^Frequency (%); other values: median (IQR)

**Fig. 4:**
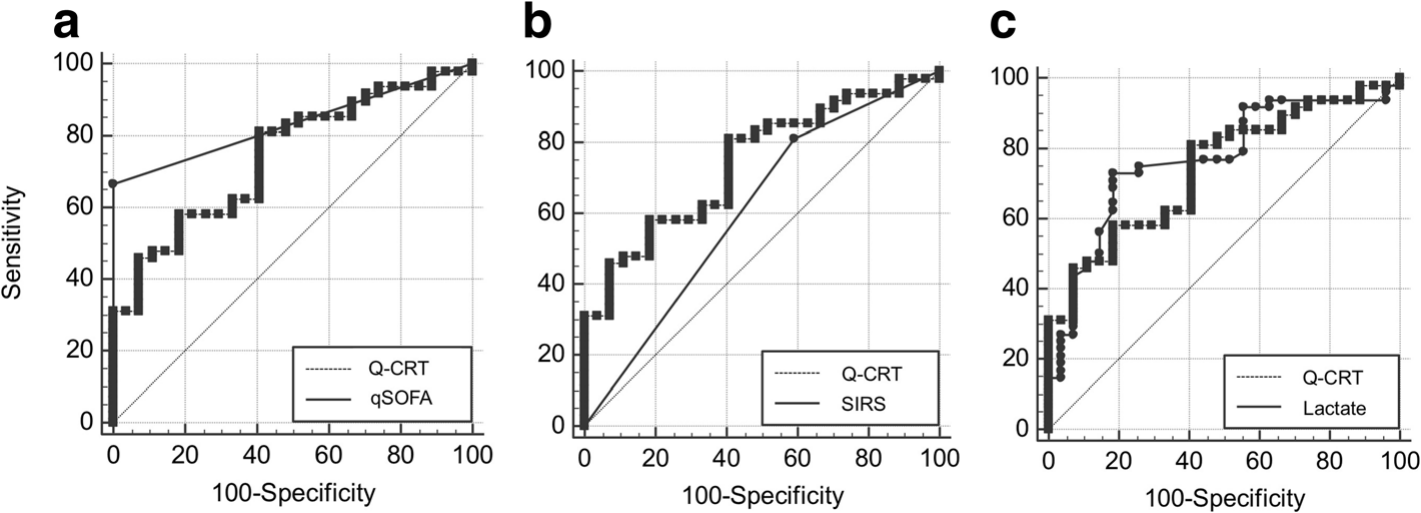
Receiver operating characteristic curves for Q-CRT, qSOFA, SIRS, and lactate level for predicting sepsis. The area under the receiver operating characteristic curve for Q-CRT is 0.71 (95% CI 0.62–0.83), that for the qSOFA score is 0.83 (95% CI 0.72–0.90), that for the SIRS score is 0.61 (95% CI 0.49–0.72), and that for the lactate level is 0.76 (95% CI 0.65–0.85). CI, confidence interval; Q-CRT, quantitative capillary refill time; qSOFA, quick sequential organ failure assessment; SIRS, systemic inflammatory response syndrome

The Q-CRT and qSOFA score were comparable as predictors of sepsis (AUC 0.74 vs. 0.83, respectively). The Q-CRT and SIRS score were also comparable as predictors of sepsis (AUC 0.74 vs. 0.61, respectively). The Q-CRT and lactate level were comparable as predictors of sepsis (AUC 0.74 vs. 0.76, respectively) (Table 2 and Fig. 4a–c). The sensitivity and specificity of the Q-CRT to predict sepsis were 0.58 and 0.81, respectively; those for the qSOFA score were 0.66 and 1.00; those for the SIRS score were 0.81 and 0.40; and those for the lactate level were 0.72 and 0.81, respectively.

**Table 2:** Performance of Q-CRT in predicting sepsis

	AUC (95% CI)	Sensitivity	Specificity	Difference between areas (95% CI)	*P* value
Q-CRT	0.741 (0.627–0.835)	58.33	81.48	–	–
qSOFA	0.833 (0.729–0.909)	66.67	100	0.0926 (−0.0384–0.224)	0.1661
SIRS	0.61 (0.490–0.721)	81.25	40.74	0.131 (−0.0118–0.273)	0.0722
Lactate	0.769 (0.658–0.859)	72.92	81.48	0.0285 (− 0.119–0.176)	0.7036

*AUC* area under the ROC curve, *CI* confidence interval, *Q-CRT* quantitative capillary refill time, *qSOFA* quick sequential organ failure assessment, *SIRS* systemic inflammatory response syndrome

**Fig. 5:**
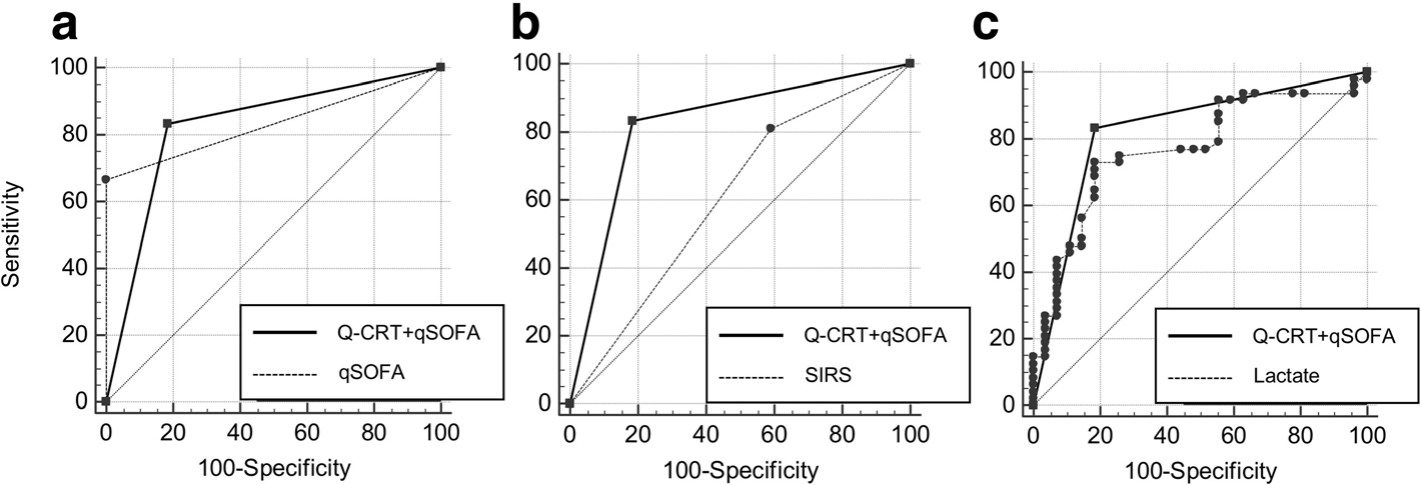
Receiver operating characteristic curves for Q-CRT + qSOFA score of ≥ 2, qSOFA, SIRS, and lactate. The area under the receiver operating characteristic curve for Q-CRT + qSOFA score of ≥ 2 is 0.82 (95% CI 0.71–0.90), that for the qSOFA score is 0.83 (95% CI 0.72–0.90), that for the SIRS score is 0.61 (95% CI 0.49–0.72), and that for the lactate level is 0.76 (95% CI 0.65–0.85). CI, confidence interval; Q-CRT, quantitative capillary refill time; qSOFA, quick sequential organ failure assessment; SIRS, systemic inflammatory response syndrome

Among patients with a Q-CRT > cutoff value + qSOFA score of ≥ 2 (Q-CRT/qSOFA combination), Q-CRT/qSOFA combination and the qSOFA score were comparable as predictors of sepsis (AUC 0.82 vs. 0.83, respectively). Q-CRT/qSOFA combination was a better predictor of sepsis than the SIRS score (AUC 0.83 vs. 0.61, respectively), and Q-CRT/qSOFA combination and lactate were comparable predictors of sepsis (AUC 0.82 vs. 0.76, respectively) (Table 3 and Fig. 5a–c). The sensitivity and specificity of Q-CRT/qSOFA combination for predicting sepsis were 0.83 and 0.81, respectively.

**Table 3:** Performance of Q-CRT + qSOFA ≥ 2 in predicting sepsis

	AUC (95% CI)	Sensitivity	Specificity	Difference between areas (95% CI)	*P* value
Q-CRT + qSOFA	0.824 (0.719–0.902)	83.33	81.48	–	–
qSOFA	0.833 (0.729–0.909)	66.67	100	0.00926 (− 0.0825 to 0.101)	0.8431
SIRS	0.61 (0.490–0.721)	81.25	40.74	0.214 (0.105 to 0.323)	0.0001
Lactate	0.769 (0.658–0.859)	72.92	81.48	0.0548 (− 0.0739 to 0.183)	0.4042

*AUC* area under the ROC curve, *CI* confidence interval, *Q-CRT* quantitative capillary refill time, *qSOFA* quick sequential organ failure assessment, *SIRS* systemic inflammatory response syndrome

## Discussion

In this study, we examined the relationship between sepsis and the Q-CRT compared with the qSOFA and SIRS scores and lactate level. We also examined the relationship between sepsis and Q-CRT/qSOFA combination compared with the qSOFA and SIRS scores and lactate level. We found that the accuracy of the Q-CRT to predict sepsis was comparable with that of the qSOFA and SIRS scores and lactate level. We also found that the accuracy of Q-CRT/qSOFA combination to predict sepsis was comparable with that of the qSOFA score and lactate level. Q-CRT/qSOFA combination was a significantly better predictor of sepsis than the SIRS score.

In our comparison of Q-CRT/qSOFA combination and the qSOFA score, the accuracy was almost identical (0.82 vs. 0.83), the sensitivity of Q-CRT/qSOFA combination was higher (0.83 vs. 0.66), and the specificity of the qSOFA score was higher (0.81 vs. 1.0). Therefore, it seems that Q-CRT/qSOFA combination is most suitable for selection of patients with sepsis for intensive care and that the qSOFA score is most suitable for selection of patients with true sepsis for analyses of sepsis.

van Genderen et al. [15] stated that the CRT is important for confirming the resuscitation status in patients with septic shock. Anderson et al. [16] reported that the normal upper limit of the CRT is 3.5 s; however, a wide range of normal values have been reported. Anderson et al. [16] also reported that age, sex, ambient temperature, and body temperature were statistically significant predictors of the CRT; altogether, however, these factors explained only 8% of the observed variability. In the present study, the cutoff value for the Q-CRT was 3.47 s with a sensitivity of 58% and specificity of 81%. The combined use of the Q-CRT cutoff with a qSOFA score of ≥ 2 had a sensitivity of 83% and specificity of 81%. In patients with sepsis, the AUC of Q-CRT/qSOFA combination was 0.82, and the AUC of the qSOFA score alone was 0.83; thus, the accuracy was comparable. By the new definition, the qSOFA score is reportedly superior to the SOFA score and SIRS score for prediction of mortality rates in non-ICU settings [17, 18]. Freund et al. [19] stated that a qSOFA score of < 2 is associated with lower mortality and that the qSOFA score has a smaller risk of overlooking severely ill patients and is a useful substitute for the SIRS score. Gu et al. [20] reported that early detection of sepsis is important to decrease the mortality rate, and our results indicate that the Q-CRT is useful for early detection of sepsis in the emergency department.

Some authors consider the sensitivity of qSOFA too low to be useful as a tool to predict sepsis [21, 22]. Fang et al. [23] retrospectively analyzed data from patients with infection in the ICU and compared the characteristic features of Sepsis-1 and Sepsis-3. Nearly 20% (18.4%) of patients diagnosed with sepsis based on Sepsis-1 did not meet the Sepsis-3 criteria. Furthermore, 139 patients (6.39%) died acutely (within 21 days). In contrast, only 6.0% of patients diagnosed with sepsis based on Sepsis-3 did not meet the Sepsis-1 criteria, and only 59 patients (9.11%) died acutely. Thus, the relative risk of overlooking severely ill patients with a poor prognosis is higher using the Sepsis-3 criteria [23].

In this study, Q-CRT/qSOFA combination had better sensitivity than the qSOFA score alone and better specificity than the SIRS score alone. There was no significant difference in the accuracy of Q-CRT/qSOFA combination and the qSOFA score or lactate concentration. The ability of the Q-CRT to predict sepsis may be similar to that of the qSOFA score or serum lactate concentration; therefore, measurement of the Q-CRT may be an alternative for invasive measurement of the blood lactate concentration in evaluating patients with suspected sepsis.

Our results showed that the creatinine level, lactate level, and Q-CRT were higher and that the platelet count was lower in patients with sepsis. Blood tests are necessary to measure parameters other than the Q-CRT. In this regard, the Q-CRT could also permit faster patient treatment because it can be obtained in an emergency setting without invasive tests, such as blood tests.

Our study has several limitations. First, the outcome measure was not the mortality rate, but whether the patient had sepsis. Second, not all patients underwent Q-CRT measurement. The study may have been subject to selection bias and documentation and data entry errors. Third, we did not verify interobserver and intraobserver reliability (interobserver and intraobserver measurement errors) of the Q-CRT. Finally, the Q-CRT has been tested and used in Japanese hospitals; therefore, studies of the device in other countries might be needed to obtain generalizable results.

## Conclusions

In this study, Q-CRT/qSOFA combination had better sensitivity than the qSOFA score alone and better specificity than the SIRS score alone. There was no significant difference in accuracy between Q-CRT/qSOFA combination and the qSOFA score or lactate concentration. The ability of the Q-CRT to predict sepsis may be similar to that of the qSOFA score or serum lactate concentration; therefore, measurement of the Q-CRT may be an alternative for invasive measurement of the blood lactate concentration in evaluating patients with suspected sepsis.

## Additional files

Additional file 2: Q-CRT data from patients with suspected infection (XLSX 34 kb)

## Abbreviations

AUC: Area under the curve
CRT: Capillary refill time
ICU: Intensive care unit
Q-CRT: Quantitative capillary refill time
qSOFA: Quick sequential organ failure assessment
SIRS: Systemic inflammatory response syndrome
SOFA: Sequential organ failure assessment
